# Influence of Adding Chinese Yam (*Dioscorea opposita* Thunb.) Flour on Dough Rheology, Gluten Structure, Baking Performance, and Antioxidant Properties of Bread

**DOI:** 10.3390/foods9030256

**Published:** 2020-02-28

**Authors:** Qing-Ming Li, Yan Li, Jin-Hao Zou, Shi-Yin Guo, Feng Wang, Peng Yu, Xiao-Jun Su

**Affiliations:** 1 College of Food Science and Technology, Hunan Agricultural University, Changsha 410128, China; 2 Hunan Provincial Research Center of Engineering and Technology for Fermented Food, Changsha 410128, China; 3 College of Science, Hunan Agricultural University, Changsha 410128, China; pengy7505@hunau.net

**Keywords:** yam, bread, gluten structure, antioxidant property, rheology

## Abstract

Impacts of wheat flour substituted with various levels of Chinese yam (*Dioscorea opposita* Thunb.) flour (from 0% to 25%) on the dough rheological characteristics, gluten structure, baking performance, and antioxidant properties of bread were investigated. The water absorption increased significantly (*p* < 0.05), while development time and stability decreased remarkably (*p* < 0.05) as the proportion of yam flour increased. SEM results indicated that the addition of yam flour destroyed the gluten network structure in the dough. Fourier Transform Infrared Spectroscopy (FTIR) spectra showed that addition of yam flour decreased the content of α-helix and β-sheet in gluten. With the increase in the proportion of yam flour, the specific volume and overall acceptability decreased (*p* < 0.05) whereas the total phenolics content (TPC), polysaccharides content, total flavonoids content (TFC), allantoin content, The 1,1-diphenyl-2-picrylhydrazyl (DPPH) radical scavenging capability, fractal dimension, and hardness increased (*p* < 0.05). Overall, breads made of wheat flour replacement with no more than 15% Guihuai number 2 yam flour were of a high quality and had more antioxidant properties. These showed that Guihuai number 2 had broad application prospects in baked products.

## 1. Introduction

Chinese yam (*Dioscorea opposita* Thunb.) has long been considered a food that improves health. In China, it is a traditional Chinese herbal medicine. Due to its high protein and starch content, yam is an important staple food in many subtropical and tropical regions and is the main source of carbohydrates and proteins [1]. Fresh yams are perishable and not easy to store. Processing of yam tubers into flour is the main mean to increase its economic value. Yam flour can be used as a food ingredient, which will broaden the utilization of yam in food processing. Yam flour has been used to prepare many traditional foods, such as amara, noodles, pasta, and baby food [2,3,4]. Yam flour has outstanding potential for making bread as a result of its high nutritional value and good processing properties [5,6,7,8]. Compared with wheat flour, yam flour is abundant in polysaccharide, phenolic, flavonoid, and allantoin compounds, and has high antioxidant ability [9,10]. Previous studies have reported that yam and wheat notably affect the functional properties and quality of bread varied with the level of substitution. Hsu et al. [6] and Liu et al. [11] reported that wheat substitution with yam flour resulted in bread with high antioxidant ability and low digestibility. Hsu et al. [6] concluded that breads made with no more than 20% yam flour did not have significant negative effects on the bread quality. Gluten makes an important impact on bread making, but yam flour lacks gluten [12]. Understanding the influence of yam flour on the structure of gluten is necessary to optimize the recipe and process of yam bread. However, there is no report on how the addition of yam flour affects the changes in gluten structure.

Guihuai number 2 is one of the yam cultivars selected by Cash Crops Research Institute, Guangxi Academy of Agricultural Sciences (Nanning, China) which has been widely planted for food use in south China. Compared with other yam cultivars, Guihuai number 2 has the characteristics of high yield, small starch molecular weight, high amylose content, and low pasting temperature. Guihuai number 2 has not been fully utilized and well-studied compared to other yam cultivars. Evaluation of Guihuai number 2 for bread making is beneficial to broaden applications and thus promote the cultivation of yam. Therefore, it is necessary to evaluate the potential applicability of Guihuai number 2 as a partial substitution for wheat flour in bread making.

Thus, the objectives of this study were to evaluate the effect of bread making substituted with Guihuai number 2 yam flour. The dough rheological properties and gluten protein structure were studied using farinograph, extensograph, rheometer, rapid visco analyzer (RVA), texture analyzer, scanning electron microscopy (SEM) and fourier transform infrared spectroscopy (FTIR). In addition, the bioactive components and antioxidant properties of bread were also investigated.

## 2. Materials and Methods

### 2.1. Materials

Wheat flour was produced by Guangzhou Grain Enterprise Group (Guangzhou, China). The composition analysis results as following (dry basis): 13.34% ± 0.04% moisture, 14.20% ± 0.10% protein, 69.87% ± 0.43% starch, 1.70% ± 0.05% fat, and 0.73% ± 0.02% ash content. Fresh yams (Guihuai number 2) were harvested from Baise city, Guangxi province. The yam tubers were washed, peeled, and sliced to 3mm thickness pieces. Yam slices were dried in a 101-2AB hot air dryer oven (Tianjin Taisite Instrument Co., Ltd, Tianjin, China) at 60 °C [13]. After drying, the slices were pulverized using a hammer (SF-130, Zhongnan Pharmaceutical Machinery Factory, Changsha, China) and sieved via a mesh of 250 μm. The proximate composition of yam flour was as following (dry basis): 6.77% ± 0.06% moisture, 12.76% ± 0.13% protein, 62.55% ± 0.33% starch, 0.56% ± 0.03% fat, and 3.95% ± 0.07% ash content. Salt, sugar, instant dry yeast (Angel Yeast Co. Ltd, Chengdu, China), and shortening (Anchor, Auckland, New Zealand) were purchased from METRO supermarket in Changsha, China. All other chemicals and reagents were of analytical grade unless otherwise stated.

### 2.2. Dough Preparation and Laboratory Bread Making Method

The method described by Nindjin, Amani, and Sindic [7] was used in bread making. The bread recipe was described as follows: 100 g of wheat flour, 0.7 g of instant dry yeast, 8 g of shortening, 18 g of sugar, 1 g of salt, and 55 g of water. Wheat flour was substituted with 0% (W100Y0), 5% (W95Y5), 10% (W90Y10), 15% (W85Y15), 20% (W80Y20), or 25% (W75Y25) yam flour weight.

### 2.3. Dough Properties

#### 2.3.1. Farinograph and Extensibility

Farinograph and extensibility were performed on farinograph and extensograph (Brabender E, Duisburg, Germany) respectively, with the method described by Li [3] and Nindjin [7].

#### 2.3.2. Texture Properties

The dough was cut into 50 × 40 × 15 mm cubes. Texture properties of dough were measured using texture profile analysis (TPA) on a TA.XT plus texture analyzer (Stable Micro Systems, Surrey, UK) with a P100 probe. The detailed condition was described in a previous study with slight modification [14]. Measurement conditions: the pre-test speed (2.0 mm/s), test and post-test speed (1.0 mm/s), and strain (65%).

#### 2.3.3. Rheological Analysis

Dough rheological analysis was performed on a Kinexus Pro+ rheometer (Malvern Instruments, Worcestershire, UK). Measurement conditions: parallel-plate geometry (20 mm diameter) with 1 mm gap, frequency range of 0.1–20 Hz and a temperature of 25 ℃. Furthermore, elastic modulus (*G*′), viscous modulus (*G*″), and loss tangent (tan *δ* = *G*″/*G*′) were also determined [14].

#### 2.3.4. Pasting Properties

The pasting analysis was performed on RVA (Model: RVA-S/N2112681, Perten, Stockholm, Sweden). The method was presented in previous reports [15,16]. Mixed flour samples (3.5 ± 0.01 g) were transferred into the canister where 25 mL of distilled water was added. The suspension was heated from 50 to 95 °C at a rate of 12 °C/min, maintained at 95 °C for 2.5 min, followed by cooling to 50 °C at a rate of 12 °C/min with another 2 min holding time.

### 2.4. Structure Analysis of Dough and Gluten

#### 2.4.1. Drying of Dough and Gluten

Water-washed gluten was prepared by using the procedure of Tuhumury et al. [17]. The wet gluten and dough were freeze-dried using a freeze-dryer (FD-1-50, Beijing Biocool Experimental Instrument Co. Ltd, Beijing, China).

#### 2.4.2. Scanning Electron Microscopy (SEM)

Micrographs of dough and gluten were visualized by a JSM-6380LV SEM (JEOL Co., Tokyo, Japan). The freeze-dried dough and gluten samples were cut into 10 × 10 × 10 mm cubes. The samples were fixed, coated with sputtered gold and examined under SEM at an energy of 25 kV.

#### 2.4.3. Fourier Transform Infrared Spectroscopy (FTIR)

FTIR measurements of gluten were performed on an IRAffinity-1 spectrometer (Shimadzu, Kyoto, Japan). The absorbance spectra was recorded between 4000–400 cm^−1^, at a resolution of 4 cm^−1^, and 32 scans. Baseline correction was performed on the amide I band (1600–1700 cm^−1^) using peakfit 4.12 software (SeaSolve Software Inc., Framingham, MA, USA). The Savitsk–Golay function was used for smoothing and deconvolution. The relative percentage of each secondary structure was calculated [18].

### 2.5. Evaluation of Bread Quality

#### 2.5.1. Bread Image Analysis

Bread was cooled at room temperature for 2 hours and then cut into 12.5 mm thick slices. The crumb images were taken with a color camera. The crumb characteristics were analyzed by the method described by Gao et al. [19] and Farrera-Rebollo et al. [20].

#### 2.5.2. Specific Volume (SV)

Cooled bread was accurately weighed, and volume was determined by the millet replacement method. The specific volume (mL/g) was calculated from the ratio of volume and mass of bread.

#### 2.5.3. Crumb Texture

Crumb texture was performed on a TA-XT2 texture analyzer (Stable Microsystems, Surrey, UK). Strain was 70% [21], other conditions were the same as Section 2.3.2.

#### 2.5.4. Antioxidant Property

Moisture content was determined by heating sample at 105 °C for 4 hours in a 101-2AB hot air dryer oven (Tianjin Taisite Instrument Co. Ltd., Tianjin, China). Fresh bread was used for test and the extract methods were described by Hsu et al. [1,6]. Total polyphenol content (TPC) and total flavonoid content (TFC) were analyzed according to the analytical procedure described by Chiu [9] and Chen [22], the results were expressed as mg gallic acid equivalent (GE)/g and rutin equivalent (RE)/g of bread sample (dry basis ), respectively. Allantoin was determined on an Agilent 1260 HPLC with a C18 column (150 mm × 4.6 mm, 5 μm). The conditions were described by Fu, Ferng and Huang [10]. Measurement of polysaccharides content was conducted on the basis of the method reported by Yang et al. [23]. The results were expressed as mg/g of bread sample (dry basis). The 1,1-diphenyl-2-picrylhydrazyl (DPPH) scavenging activity of bread was measured using the procedure described by Hsu et al. [6]. The EC50 value represented the effective concentration when 50% of radicals were scavenged.

#### 2.5.5. Sensory Evaluation

The sensory panelists (5 males and 5 females; age 20–40) were recruited based on their previous experience in the sensory evaluation of baked foods among postgraduate students and teachers of the Hunan Provincial Research Center of Engineering and Technology for Fermented Food, China. Sensory evaluation was determined according to a nine point hedonic rating scale method, the analytical procedure as described by Nindjin [7].

### 2.6. Statistical Analysis

All trials were carried out in triplicate and the data expressed by average ± standard deviation (SD) were analyzed by SPSS 22.0 (IBM, Chicago, IL, USA) and statistical significance was determined at 0.05 levels (*p* ≤ 0.05).

## 3. Results and Discussion

### 3.1. Effect of Yam Flour Substitution on Pasting, Farinograph, Rheological, and Texture Properties of Dough

The dough properties were evaluated using RVA, farinograph, extensograph, and texture analyzer, and the results were listed in Table 1 and Table 2. Rheological properties were closely related to the baking ability of the dough, which was an important index for evaluating the baking performance. Substitution of wheat flour with yam flour had remarkable effects on pasting properties (Table 1). Substitution of yam flour led to a remarkable (*p* < 0.05) decrease of trough viscosity (from 1540 to 350 cP (1 cP = 10^−3^ Pa·s)), final viscosity (from 2737 to 745 cP), peak viscosity (from 2275 to 1285 cP), peak time (from 6.60 to 5.02 min) and setback (from 1198 to 395 cP), while the overall trend of breakdown showed remarkable (*p* < 0.05) increase. The decrease of viscosity values could be related to the composition, starch structure and mucilage of yam. Similar trends were reported that the viscosity values were significantly decreased by purple yam flour [3], purple sweet potato flour [24], and soybean flour [25] addition. An increase in breakdown indicated a decrease in the thermal stability of the mixed flour, which may be related to the amylose content. Addition of yam flour decreases the setback of the mixed flour (*p* < 0.05). This is due to amylose–amylopectin ratio changes in these mixed flours, which had impact on the crumb structure and the specific volume of bread [14].

The farinographic analysis could reflect the rheological properties of the dough during its formation [26]. After adding yam flour, farinograph properties of dough changed significantly (*p* < 0.05). According to Nindjin et al. [7], yam starch caused a decrease in water absorption, development time and dough stability. On the contrary, this result showed that increasing the addition of yam flour, the water absorption was increased. Similar findings were observed in purple yam flour [3], potato flour [27]. The increase in water absorption of the mixed flour may be due to the yam flour containing a highly absorbent mucilage. Development time of the dough reflects the quantity and quality of gluten. The higher the quantity and quality of gluten, the longer the development time. Development time and dough stability of different yam flour proportions were remarkably decreased. The softening degree of W100Y0 was the lowest, which was increased significantly with yam flour addition. Because gluten is not present in yam flour, mixed flour dough could not develop a strong gluten network, which reduced the cohesivity and elasticity of the dough. Liu [27] reported that water absorption and degree of softening increased with the increasing addition of potato flour in mixed flour (10%, 15%, 20%, 25%, 30%, and 35%).

The effect of yam addition on extensographic properties after 135 min was also outlined in Table 1. The area under the curve, extensibility, resistance to extension, and max-extensible resistance of the dough showed a significant downward trend with increasing yam flour proportion from 0% up to 25% (*p* < 0.05). According to Li et al. [3], wheat flour substituted with 5%–20% purple yam flour had an increased resistance to extension and decreased extensibility. This may be related to the different varieties of yam. Energy area was the total energy demanded when the dough was broken down, and it was an important parameter that reflects the elasticity of the dough. A higher energy area means more dough elasticity [28]. The addition of yam flour reduced the gluten content of the yam–wheat mixed flour, resulting in a decrease in the content of gliadin and glutenin, leading to a reduction in viscoelasticity.

As showed in Table 2, In case of W95Y5 and W90Y10, the adhesiveness was similar to W100Y0 (*p* > 0.05). Compared with W100Y0, hardness and chewiness of W95Y5 were largely reduced (*p* < 0.05). Hardness, adhesiveness and chewiness of dough showed a significant tendency for increasing first and then decreasing with the addition of yam flour from 5% to 25% (*p* < 0.05). Similar trends were also reported by Belghith Fendri et al. [29]. The higher strength of dough with yam flour addition demonstrated that the bread obtained presented higher hardness.

**Figure 1:**
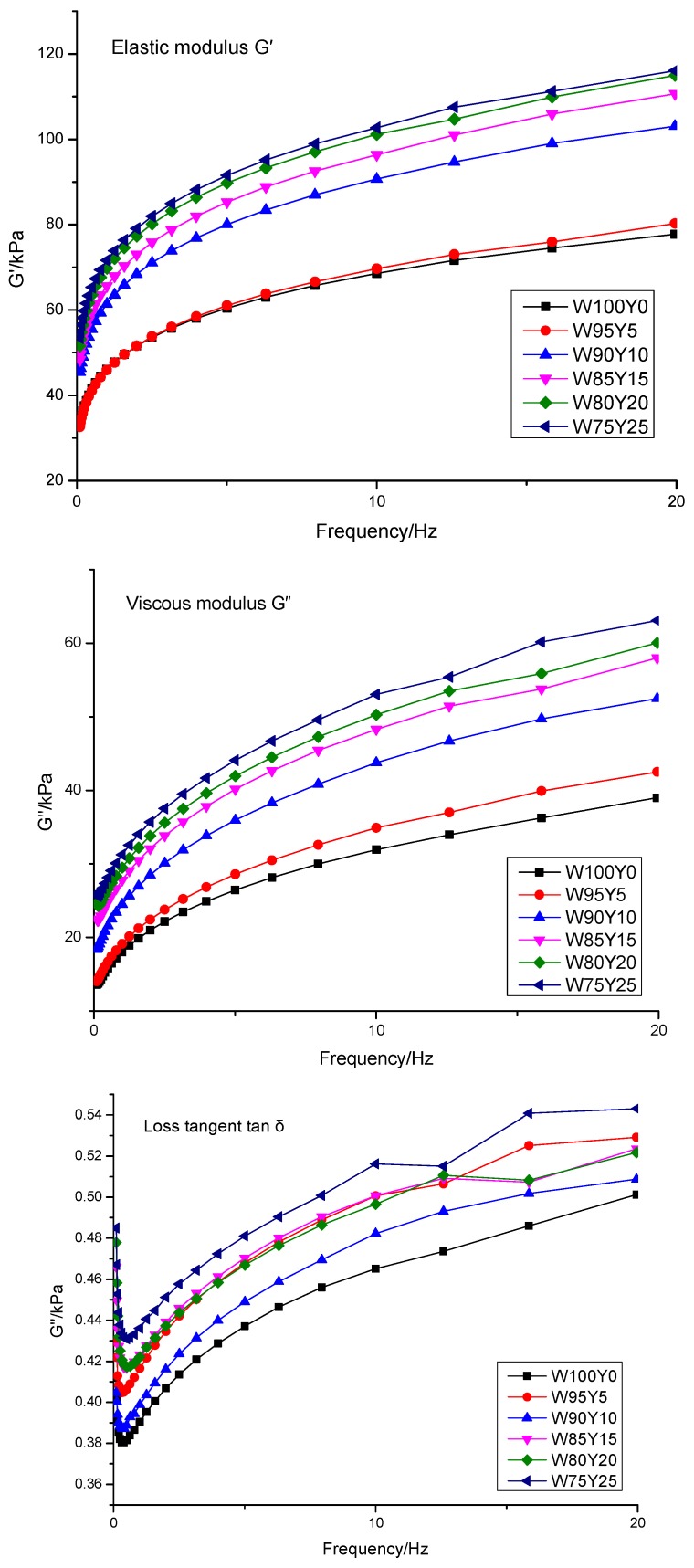
Frequency sweep data for wheat dough with different yam contents.

The frequency sweep test was conducted to analyze the rheological properties of dough. Figure 1 showed the elastic modulus (*G*′), viscous modulus (*G*″), and loss tangent (tan δ) of the dough. *G*′ was larger than *G*″ (tan δ < 1) at frequency range from 0.1 to 20 Hz. These phenomena indicated that elastic properties were predominated and the dough had solid, elastic-like features. The *G*′ and *G*″ of all dough samples increased as frequency increased. Similar trends were reported by Cao [30] and Balestra [31].

Compared to other samples, Dough W100Y0 had the lowest *G*′ and *G*″. Meanwhile, *G*′ and *G*″ increased as yam flour content increased. Dough W75Y25 showed higher *G*′ and *G*″ in comparison with dough with low level substitution of yam flour. These results indicated that substitution of yam flour significantly affected the dough structure and gluten network, and the influence became more pronounced as the amount of yam flour increased. Similar results were illustrated in studies of adding potato flour [27]. Furthermore, the increasing trend of *G*′ and *G*″ was different from that of Cao [30] who argued that replacement of potato pulp decreased *G*′ and *G*″. These results could be explained by the complex effects of protein, mucilage, and polysaccharides on dough viscoelasticity. During bread making, the rheological characteristics were dominated by the cross-linking of gluten network. Therefore, adding Yam flour to the dough, the gluten network had a higher crosslinking density. As shown in Figure 1, tan *δ* increased in the dough when the level of yam flour addition increased, which implied that the cross-linking degree in gluten network was reduced. Balestra [31] reported that the higher value of tan δ might be due to the lower quantity of cross-linking in gluten network. Tanδ of the dough decreased within 0.1–0.5 Hz. However, at 0.5–20 Hz, tanδ continued to increase as the frequency continued to increase, indicating that the structure of the yam-wheat mixed flour dough at higher frequencies was unstable and easily damaged.

The rheometer and texture data analyzer agreed with the farinograph results. These results revealed that the dough with yam flour substitution had a reduced capacity to keep its shape during proofing. Dough with yam flour substitution also decreased gas retention during the fermentation process, which affects bread porosity.

### 3.2. Effect of Yam Flour on the Secondary Structure of Gluten Protein

**Figure 2:**
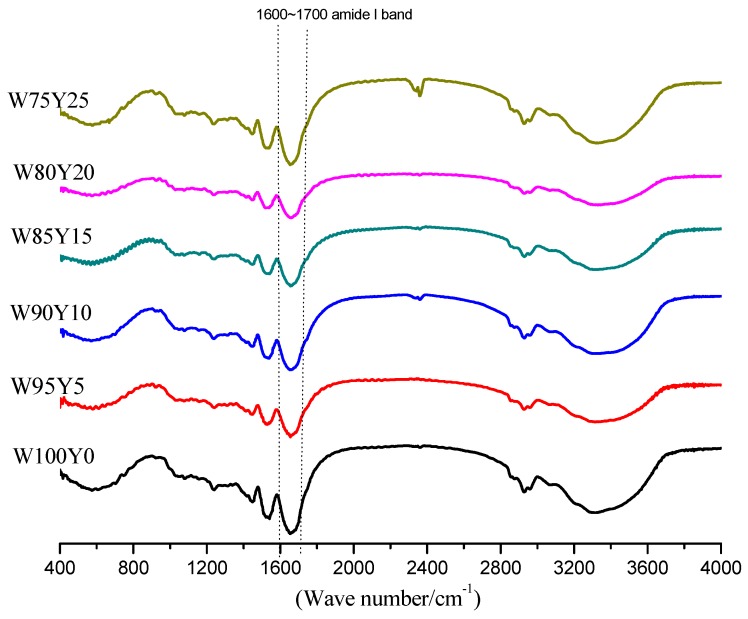
Fourier Transform Infrared Spectroscopy (FTIR) spectra of gluten protein in dough with different amounts of added yam flour.

**Figure 3:**
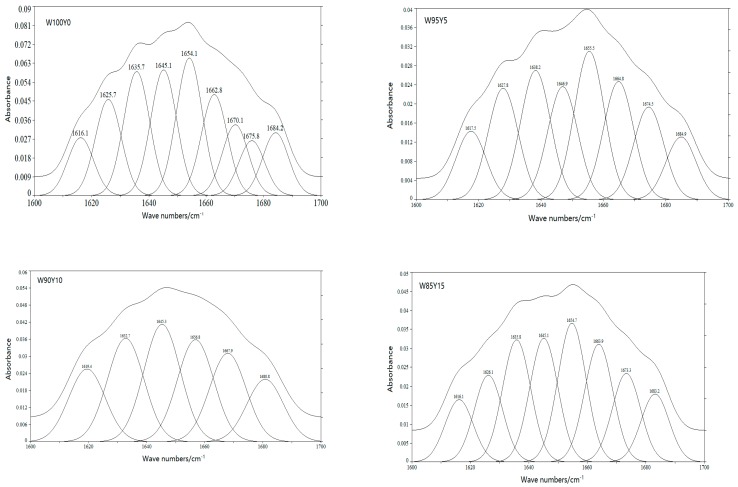

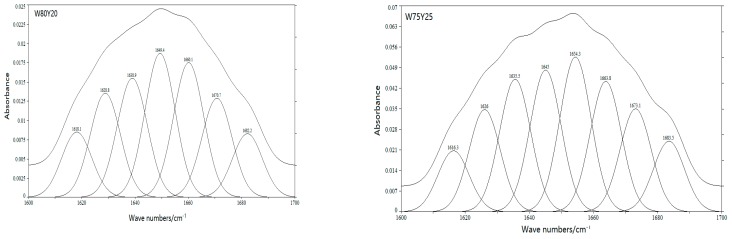
Second-derivative spectra in amide I band of gluten proteins enriched with different levels of yam flour.

FTIR spectra of gluten were measured to clarify the effect of yam flour on gluten conformation (Figure 2). The secondary structure of gluten protein includes α-helix, β-sheet, β-turn, random coil, and so on. Amide I band was often used to identify the secondary structure of protein. In the amide I band, the secondary structure corresponding to each characteristic peaks were: 1615–1637 cm^−1^ and 1682–1700 cm^−1^ were β-sheet characteristic peaks; 1637–1645 cm^−1^ was random coil characteristic peak; 1646–1664 cm^−1^ was the characteristic peak of α-helix; and 1664–1681 cm^−1^ was the characteristic peak of β-turn [32]. α-helix and β-sheet were relatively ordered protein secondary structures, while β-turn and random coil were disordered structures. The stability of α-helix and β-sheet was relatively high. Figure 3 was the characteristic curve of gluten protein after fitting in the band of 1600–1700 cm^−1^. The content of secondary structure of gluten protein was listed in Table 3.

It has been generally accepted that the most stable secondary structure was β-sheets. Similarly, an increasing in the α-helix would also lead to a more ordered structure [33]. According to Table 3, the proportion of α-helix and β-sheet in W100Y0 gluten protein was 73.26%, and the proportion of random coil was 15.13%. The secondary structure of gluten protein was dominated by β-sheet, which is consistent with previous reports [34]. After adding yam flour, the percentage of α-helix and β-sheet in gluten was lower than that in W100Y0, while the proportion of random coil was higher than W100Y0. A similar regularity was demonstrated by Chen [12]. The decrease in the proportion of ordered structures and the increase in the proportion of disordered structures in gluten protein were consistent with the results of dough rheological properties.

### 3.3. Effect of Yam Flour on Antioxidant Capacity of Bread

Total phenolics contents (TPC), polysaccharides content, total flavonoids content (TFC), allantoin content and radical scavenging capability (IC_50_ Value) of bread with different substitution ratio of yam flour were showed in Table 4. As showed in Table 4, TPC, polysaccharides, TFC and allantoin of bread increased with substitution ratio dependence. These results were in good agreement with previous work [4]. Yam flour addition remarkably affected the DPPH radical scavenging capability of bread. As could be seen from Table 4, the IC50 values of W100Y0 and W95Y5 were the highest, indicating that their radical scavenging capability was the lowest. Jing and Kitts [35] reported that Maillard reaction products showed certain radical scavenging capability. It is interesting that a 10%–25% yam flour substitution caused remarkable improvement in DPPH scavenging capability. The radical scavenging capability of bread was strongly dependent on addition of yam flour. DPPH radical scavenging capability increase trend was consistent with TPC, polysaccharides, TFC, and allantoin content of bread.

### 3.4. Digital Images of Dough and Gluten

**Figure 4:**
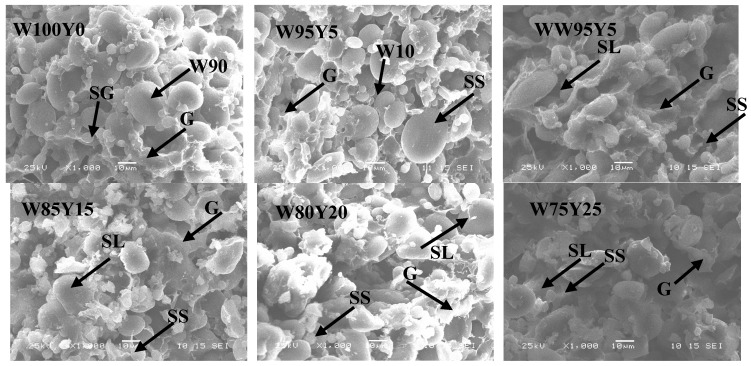
Scanning electron micrographs of dough substituted with different level of yam flour (1000×). SL: large starch granules, SS: small starch granules, and G: gluten network.

As shown in Figure 4, SEM of W100Y0 showed a typical structure of wheat dough [27]. The SEM image of dough W95Y5 was similar to W100Y0, the network structure of gluten was well-established, with continuity, the small and large starch granules were arranged tightly and evenly, and they were embedded in the network structure of gluten. These results agreed with the changes in rheological properties of dough characterized by rheometer and texture analyzer. With the increase of yam flour substitution, the continuity of gluten network structure became worse, and the ability to wrap the starch granules became lower. When the added amount was 10% (W90Y10), the gluten protein of the dough began to appear discontinuous, and the starch granules were exposed outside the gluten network. When the added amount was 15% (W85Y15), it could be seen that the starch granules obviously separated from the gluten matrix. When the addition amount was 20% or 25% (W80Y20 or W75Y25), the gluten protein content decreased, and the gluten matrix was almost invisible. The number of irregular voids increased, and the gluten structure was seriously damaged. The scanning electron microscope results further confirmed that substitution of yam flour would affect the development of continuous gluten network structure, destroyed the gluten network structure, and caused changes in dough characteristics. These trends were corresponding to previous results of reduction in dough stability and viscoelasticity of the dough with yam flour addition. Liu et al. [27] and Cao et al. [30] reported that the substitution of potato flour and potato pulp could break the gluten network, thereby changing the viscoelasticity of dough.

**Figure 5:**
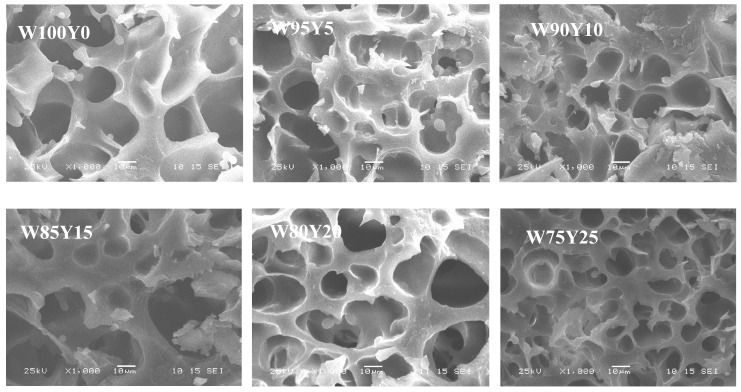
SEM images of gluten substituted with different level of yam flour. The magnifications of SEM images were 1000×.

Figure 5 was a scanning electron microscope image of gluten at a magnification of 1000 times. It can be seen from Figure 5 that W100Y0 showed a compact gluten structure with uniform pores, and the edges of pores were smooth, indicating the formation of a solid gluten structure. When yam flour was added at a low level (W95Y5), gluten protein could still maintain a good structure. As the proportion of yam flour continued to increase, gluten structure changed significantly. The smoothness and uniformity of gluten were damaged, and the degree of damage increased with the increase of ratio of yam flour. The hole edges were no longer smooth, but were jagged, and the holes structure began to collapse. When the content of yam flour reached 25% (W75Y25), the gluten protein structure was honeycomb, and the holes became noticeably smaller, suggesting a weakened gluten network structure. The changes in the network structure of gluten may be due to the competition between the mucilage in the yam flour and the water content of starch and protein, which redistributed the moisture in the dough, caused partial dehydration of the gluten protein, changed the gluten protein network structure, and even caused partial collapse. Several reports have shown that the blend of bran or salt could have a significant impact on the gluten network [12,36].

### 3.5. Effect of Yam Flour on Bread Baking Performance

**Figure 6:**
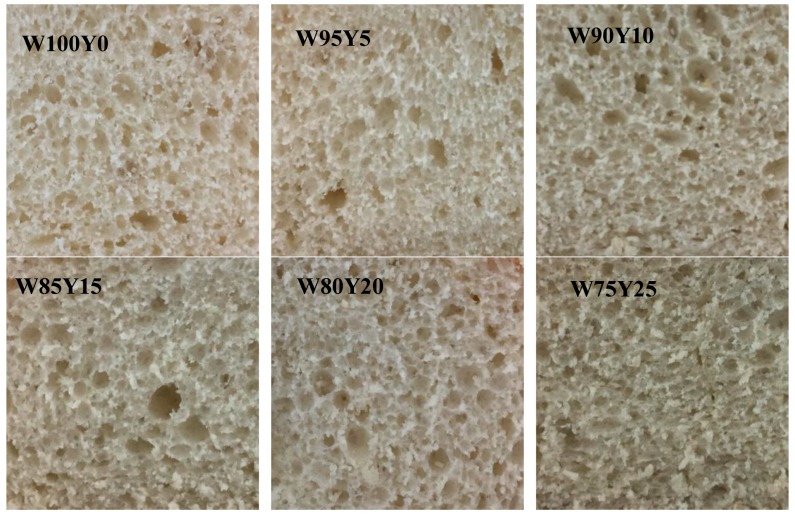
The effect of added yam flour on the characteristics of bread.

The images of bread crumb were presented in Figure 6. It can be seen from Figure 6 that with the increase of the yam flour addition ratio, the bread crumb showed more non-uniform and larger pores. In order to further analyze the structure of the bread crumb, a fractal analysis was performed on Figure 6, and the results were listed in Table 5. As the proportion of yam flour increased, the fractal dimension of bread increased. When yam flour was added at 5%, there was no remarkable difference in the fractal dimension of W95Y5 and W100Y0 (*p* > 0.05). When the addition of yam flour reached 10%, the fractal dimension was significantly higher than that of W100Y0. When the addition of yam flour was 25% (W75Y25), the fractal dimension of the bread was 1.80, which was an increase of 5.26% compared to W100Y0. The fractal dimension of the pore size distribution indicated the uniformity of the pore size distribution. The smaller the fractal dimension, the more uniform the pore size distribution. On the other hand, the larger the fractal dimension, the more uneven the pore size distribution and the greater the difference between the pore sizes. The increase of the fractal dimension indicates that the addition of yam flour changed the gas cell size formed inside the bread, and the difference in gas cell size increased. This may be due to the addition of yam flour, which affects the gas-holding properties of the dough. Crumb porosity affects the springiness and hardness of bread [37].

It could be seen from Table 5 that as the ratio of yam flour increased, the hardness of bread decreased first and then increased. When the amount of yam flour was more than 5%, the bread hardness was higher than that of W100Y0, and it increased significantly as the ratio of yam flour increased (*p* < 0.05). When the amount of yam flour was 25% (W75Y25), the bread hardness was 789.20 g, which was an increase of 67.10% compared with W100Y0. The increase in bread hardness was consistent with the change in dough hardness. Hardness had a greater effect on the overall score of bread, and had a negative correlation with bread quality. The smaller the hardness, the softer the bread. Springiness decreased remarkably with the increase in the amount of yam flour (*p* < 0.05). There was a positive correlation between the springiness and the quality of the bread. The larger the springiness, the better the quality. The decrease in bread springiness may be caused by the increased proportion of yam flour, the decreased gluten content of the mixed flour, the fact that the gluten network structure was destroyed, and the gas retention of dough was reduced. Good air holding resulted in decreased bread hardness and improved springiness. The tendency of bread cohesiveness and chewiness was consistent with hardness.

The results of the effect of yam flour addition on specific volume were presented in Table 5. The specific volume was remarkably (*p* < 0.05) affected by yam flour addition, the highest specific volume was observed in W100Y0 and the lowest was in W75Y25. As Yam flour content increased, the specific volume decreased. This showed that gas holding capacity of bread had decreased during proofing and baking. This decrease would be due to destruction of gluten by adding yam flour, in agreement with the change of gluten network. A similar trend was observed by addition of other flours such as acha and bambara nut sourdough flour [38], bran [39,40], cassava, and three leaf yam [8].

As can be seen from Table 5, the overall acceptability of bread W100Y0 was similar to W95Y5 (*p* > 0.05). When the amount of yam flour was more than 5%, there was a remarkable difference between the overall acceptability of bread and bread W100Y0 (*p* < 0.05), and it decreased gradually when the substitution of yam flour increased (*p* < 0.05). When the amount of yam flour was not more than 15%, the antioxidant activity of the bread was high and the degree of acceptance was relatively high. The main reason was that the gluten content in the yam-wheat mixed flour decreased, the formation of gluten network structure was affected and resulted in a reduction in the sensory quality of the bread. Eke-Ejiofor J. et al. [8] reported that substitution of three leaf yam flour of no more than 5% would produce bread without affecting the bread quality, while other researches showed that the yam flour addition of no more than 20% had not significant negative impact on bread quality [6,11]. This may be related to the differences in the composition of yam of different varieties.

## 4. Conclusions

Replacement of wheat flour with yam flour remarkably affected the dough characteristics and bread qualities. With the increased percentage of yam flour, the breakdown, water absorption, degree of softening, *G*′ and *G*″ increased, while the peak time, viscosity value, setback, developing time, stability, and extensibility decreased. Moreover, as the proportion of yam flour was increased, the hardness, springiness and chewiness of dough decreased first and then increased. The microstructure of dough and gluten was destroyed, and the proportion of a relatively stable structure in protein secondary structure decreased by the addition of yam flour. In addition, the specific volume, hardness, and springiness of bread were all affected by changes to dough rheological and structural properties, following the addition of yam flour. The content of polyphenols, flavonoids, polysaccharides, allantoin, and the scavenging ability of DPPH free radicals in bread showed an increase which depended on the amount of yam flour. Compared with W100Y0, the addition of 5% yam flour didn’t exert significant negative impact on overall acceptability of bread. Acceptable bread was made by the substitution of no more than 15% yam flour for wheat flour in bread recipe. Adding Guihuai number 2 yam flour to bread recipe will improve the nutritional value and antioxidant capability of bread. The results of the present study demonstrated that Guihuai number 2 had broad application prospects in baked products.

## Figures and Tables

**Table 1:** Effects of yam flour addition on pasting, farinographic, and extensographic properties of mixed flour.

Item	W100Y0	W95Y5	W90Y10	W85Y15	W80Y20	W75Y25
Pasting temperature (°C)	69.10 ± 0.05^b^	70.02 ± 0.88^ab^	69.97 ± 0.88^ab^	69.42 ± 0.51^b^	70.02 ± 0.80^ab^	71.12 ± 1.0^a^
Peak time (min)	6.60 ± 0.07^a^	5.93 ± 0.07^b^	5.58 ± 0.08^c^	5.38 ± 0.04^d^	5.31 ± 0.03^d^	5.02 ± 0.02^e^
Peak viscosity (cP)	2275 ± 52.92^a^	1441 ± 6.66^b^	1271 ± 18.33^c^	1285 ± 7.00^c^	1232 ± 2.31^d^	1285 ± 9.54^c^
Trough viscosity (cP)	1540 ± 52.92^a^	629 ± 9.45^b^	440 ± 13.53^c^	391 ± 13.23^d^	355 ± 1.73^d^	350 ± 5.51^d^
Final viscosity (cP)	2737 ± 54.60^a^	1488 ± 9.02^b^	1068 ± 12.17^c^	911 ± 23.12^d^	791 ± 10.02^e^	745 ± 25.51^e^
Setback (cP)	1198 ± 15.56^a^	859 ± 17.44^b^	628 ± 5.00^c^	520 ± 10.69^d^	436 ± 9.61^e^	395 ± 20.30^f^
Breakdown (cP)	736 ± 43.50^e^	811 ± 7.51^d^	831 ± 25.51^c^	894 ± 9.17^b^	877 ± 0.58^b^	935 ± 8.08^a^
Water absorption (%)	63.4 ± 0.00^d^	63.6 ± 0.06^c^	64.5 ± 0.10^b^	65.4 ± 0.06^a^	65.5 ± 0.10^a^	65.4 ± 0.10^a^
Dough development time (min)	2.9 ± 0.06^a^	2.2 ± 0.06^b^	1.9 ± 0.10^c^	1.7 ± 0.00^d^	1.7 ± 0.06^d^	1.7 ± 0.06^d^
Dough stability (min)	18.1 ± 0.20^a^	15.3 ± 0.35^b^	15.0 ± 0.20^b^	1.5 ± 0.06^c^	1.5 ± 0.10^c^	1.5 ± 0.06^c^
Degree of softening (FU)	22 ± 0.58^e^	35 ± 1.53^d^	44 ± 2.52^c^	86 ± 2.65^a^	76 ± 3.06^b^	88 ± 2.00^a^
Area under the curve (energy, cm^2^)	114 ± 4.58^a^	82 ± 3.06^c^	93 ± 4.00^b^	68 ± 1.00^d^	66 ± 4.16^d^	58 ± 2.52^e^
Extensibility (mm)	108 ± 5.03^a^	89 ± 5.69^b^	105 ± 6.11^a^	83 ± 3.78^bc^	80 ± 4.51^bc^	76 ± 2.00^c^
Resistance to extension (EU)	878 ± 20.52^a^	843 ± 25.36^a^	831 ± 30.62^a^	736 ± 31.90^b^	713 ± 22.05^b^	634 ± 44.96^c^
Max-extensible resistance (EU)	944 ± 46.52^a^	858 ± 28.10^b^	844 ± 23.90^b^	737 ± 15.52^c^	733 ± 14.53^c^	658 ± 31.97^d^
Extensible rate	8.16 ± 0.35^bc^	9.53 ± 0.57^a^	7.90 ± 0.18^c^	8.91 ± 0.25^ab^	8.90 ± 0.64^ab^	8.35 ± 0.48^bc^
Max-extensible rate	8.77 ± 0.38^ab^	9.69 ± 0.30^a^	8.04 ± 0.68^b^	8.93 ± 0.58^ab^	9.15 ± 0.69^a^	8.67 ± 0.60^ab^

The values (mean ± standard deviation) followed by the same letter in the same row were not significantly different (*p* > 0.05) as determined by Duncan’s multiple range test (*n* = 3).

**Table 2:** Effects of yam flour addition on texture properties of mixed flour dough.

Sample	Hardness (g)	Adhesiveness (g·s)	Chewiness (g)
W100Y0	4891.46 ± 282.38^c^	5129.16 ± 398.46^d^	3932.19 ± 226.28^c^
W95Y5	3919.22 ± 157.88^d^	5328.83 ± 242.85^d^	3121.39 ± 193.17^d^
W90Y10	5084.16 ± 121.99^c^	5016.86 ± 301.85^d^	4031.96 ± 144.53^c^
W85Y15	5712.79 ± 475.73^b^	5709.71 ± 86.72^c^	4543.46 ± 318.80^b^
W80Y20	6722.81 ± 334.47^a^	7440.66 ± 351.81^a^	5551.42 ± 239.90^a^
W75Y25	5537.48 ± 178.36^b^	6356.22 ± 246.05^b^	4622.98 ± 189.89^b^

The values (mean ± standard deviation) followed by the same letter in the same column were not significantly different (*p* > 0.05) as determined by Duncan’s multiple range test (*n* = 3).

**Table 3:** Secondary structure of gluten protein in wheat dough enriched with different amounts of yam flour.

Sample	Relative Proportion (%)			
	α-Helix	β-Sheet	β-Turn	Random Coil
W100Y0	32.24	41.02	6.61	15.13
W95Y5	30.98	28.75	24.97	15.30
W90Y10	18.67	32.34	27.61	21.48
W85Y15	31.75	41.98	10.96	15.31
W80Y20	38.18	31.93	13.57	16.32
W75Y25	31.82	40.88	11.49	15.81

**Table 4:** Total phenolics content (TPC), polysaccharides, total flavonoids content, allantoin content, and IC50 values of bread with different levels of yam flour.

Sample	Total Phenolics Content (mg GE/g)	Polysaccharides Content (mg/g)	Total Flavonoids Content (mg RE/g)	Allantoin Content (mg/g)	IC_50_Value (mg/mL)
W100Y0	0.467 ± 0.002^d^	21.18 ± 0.14^f^	0.162 ± 0.004^e^	0.05 ± 0.011^f^	124.14 ± 1.62^a^
W95Y5	0.470 ± 0.002^cd^	22.45 ± 0.06^e^	0.268 ± 0.006^d^	0.279 ± 0.006^e^	130.09 ± 1.44^a^
W90Y10	0.471 ± 0.002^cd^	25.04 ± 0.11^d^	0.378 ± 0.008^c^	0.459 ± 0.066^d^	117.36 ± 0.82^b^
W85Y15	0.480 ± 0.003^b^	27.65 ± 0.04^c^	0.475 ± 0.003^b^	0.781 ± 0.012^c^	113.37 ± 0.47^c^
W80Y20	0.481 ± 0.002^b^	29.67 ± 0.09^b^	0.525 ± 0.015^a^	0.894 ± 0.004^b^	102.98 ± 0.95^d^
W75Y25	0.491 ± 0.002^a^	31.52 ± 0.07^a^	0.516 ± 0.009^a^	1.063 ± 0.001^a^	101.36 ± 0.95^d^

The values (mean ± standard deviation) followed by the same letter in the same column were not significantly different (*p* > 0.05) as determined by Duncan’s multiple range test (*n* = 3).

**Table 5:** Effects of yam flour addition on texture properties of bread.

Sample	Fractal Dimension	Hardness (g)	Springiness	Cohesiveness	Chewiness (g)	Specific Volume (mL/g)	Overall Acceptability
W100Y0	1.71 ± 0.01^c^	472.32 ± 29.31^de^	0.639 ± 0.035^a^	0.567 ± 0.008^a^	170.90 ± 10.09^b^	4.47 ± 0.02^a^	98.0 ± 0.67^a^
W95Y5	1.72 ± 0.01^c^	422.87 ± 15.41^e^	0.618 ± 0.012^ab^	0.541 ± 0.019^b^	141.53 ± 12.37^c^	4.26 ± 0.06^b^	96.7 ± 1.64^a^
W90Y10	1.74 ± 0.01^b^	486.52 ± 23.30^cd^	0.590 ± 0.020^bc^	0.521 ± 0.010^bc^	149.41 ± 9.75^c^	4.06 ± 0.04^c^	92.2 ± 1.26^b^
W85Y15	1.75 ± 0.00^b^	530.83 ± 33.63^bc^	0.580 ± 0.005^c^	0.505 ± 0.011^c^	155.38 ± 10.24^bc^	3.97 ± 0.01^d^	89.1 ± 1.84^c^
W80Y20	1.76 ± 0.00^b^	575.94 ± 20.40^b^	0.582 ± 0.015^c^	0.510 ± 0.019^c^	171.22 ± 13.18^b^	3.89 ± 0.06^d^	80.8 ± 3.38^d^
W75Y25	1.80 ± 0.01^a^	789.20 ± 49.57^a^	0.586 ± 0.004^bc^	0.476 ± 0.010^d^	220.04 ± 12.24^a^	3.80 ± 0.07^e^	76.1 ± 2.23^e^

The values (mean ± standard deviation) followed by the same letter in the same column were not significantly different (*p* > 0.05) as determined by Duncan’s multiple range test (*n* = 3).
